# Supplementary material for: Retrospective Monocentric Clinical Study on Male Infertility: Comparison between Two Different Therapeutic Schemes Using Follicle-Stimulating Hormone
Source: J Clin Med. 2021 Jun 17;10(12):2665. doi: 10.3390/jcm10122665 (PMC8233778; doi:10.3390/jcm10122665)
Supplement: Supplementary file 1 [file jcm-10-02665-s001.zip › jcm-1250289-supplementary.pdf]

Journal of
Clinical Medicine

MDPI

Supplementary Table S1. Multilinear regression.

Post-treatment total sperm count – Group A

Independent variables	*Coeff*	*SE*	*t*	*p*	*VIF*
Constant	65.73				
Age	0.25	0.87	0.29	0.77	1.13
BMI	-1.46	2.02	-0.72	0.48	1.37
FSH*	5.09	4.75	1.07	0.30	1.21
TT*	0.02	0.10	0.26	0.80	1.25
Testicular volume*	-4.38	3.86	-1.13	0.27	1.50
Total sperm count*	0.93	0.53	1.74	0.10	1.27

Post-treatment percentage of sperm fragmented DNA – Group A

Independent variables	*Coeff*	*SE*	*t*	*p*	*VIF*
Constant	-5.80				
Age	0.00	0.07	0.00	0.10	1.22
BMI	0.10	0.16	0.63	0.54	1.20
FSH*	0.34	0.40	0.87	0.40	1.23
TT*	0.00	0.01	0.47	0.64	1.20
Testicular volume*	0.33	0.31	1.05	0.31	1.44
DNA fragmentation*	0.23	0.14	1.67	0.11	1.37

Post-treatment total sperm count – Group B

Independent variables	*Coeff*	*SE*	*t*	*p*	*VIF*
Constant	-29.16				
Age	0.25	0.98	0.25	0.80	1.61
BMI	1.40	1.62	0.86	0.40	1.26
FSH*	0.34	4.06	0.08	0.93	1.32
TT*	-0.01	0.08	-0.15	0.88	1.88
Testicular volume*	-0.82	2.78	-0.30	0.77	2.09
Total sperm count*	1.56	0.50	3.12	0.01	1.25

Post-treatment percentage of sperm fragmented DNA – Group B

Independent variables	*Coeff*	*SE*	*t*	*p*	*VIF*
Constant	2.65				
Age	-0.04	0.10	-0.37	0.71	1.61
BMI	-0.01	0.19	-0.08	0.94	1.65
FSH*	-0.36	0.44	-0.83	0.42	1.34
TT*	-0.00	0.01	-0.05	0.96	2.06
Testicular volume*	0.37	0.33	1.10	0.29	2.64
DNA fragmentation*	0.22	0.19	1.17	0.26	1.82

*values at baseline

Abbreviations. BMI= body mass index; FSH= follicle-stimulating hormone; TT= total testosterone
